# The ultrasonographic medullary “rim sign” versus medullary “band sign” in cats and their association with renal disease

**DOI:** 10.1111/jvim.15878

**Published:** 2020-09-05

**Authors:** Alessia Cordella, Pascaline Pey, Francesco Dondi, Marilyn Dunn, Chiara Caramazza, Mario Cipone, Alessia Diana

**Affiliations:** ^1^ Department of Veterinary Medical Science, Alma Mater Studiorum University of Bologna Ozzano Emilia (BO) Italy; ^2^ Department of Clinical Sciences, Faculty of Veterinary Medicine University of Montréal Saint‐Hyacinthe QC Canada; ^3^ Present address: Department of Medical Imaging of Domestic Animals, Faculty of Veterinary Medicine Ghent University Merelbeke Belgium; ^4^ Present address: Concordia Veterinary Clinic, Portogruaro Venezia Italy

**Keywords:** feline, kidney, medulla, ultrasound

## Abstract

**Background:**

Medullary rim sign (MRS) refers to a hyperechoic line in the renal medulla, reported on ultrasound examination (US) in both dogs and cats with and without kidney disease (KD).

**Objective:**

To describe the different aspects of MRS in cats and to assess its association with KD.

**Animals:**

Cats that underwent US examination, with MRS (study group) with and without KD and without MRS with and without KD (control groups).

**Methods:**

Retrospective case‐control study: cats with MRS, with or without KD (rim sign groups) and cats without MRS, with or without KD (control groups). Ultrasonographic images were blindly reviewed with attention given to the thickness and margins of the MRS recorded.

**Results:**

Eighty‐four cats with MRS were included and 60 cats recruited for each control group. The MRS had 2 distinct aspects: a thin hyperechoic line with well‐defined margins (MRS‐*line*) in 50/84 cats (59%) and a thick hyperechoic band with ill‐defined margins (MRS‐*band*) in 34/84 cats (41%). Twenty of 50 (40%) cats with MRS‐*line* and 25/34 (74%) of cats with MRS‐*band* had KD. The frequency of MRS‐*line* was higher in cats without KD, whereas the presence of MRS‐*band* was more frequent in cats with KD (*P* = .003).

**Conclusions and Clinical Importance:**

A thick hyperechoic ill‐defined band (for which the term medullary band sign is proposed) was more frequently associated with KD, whereas a thin hyperechoic well‐defined line (true MRS) may be seen in cats with or without KD.

**Abbreviations**
AUC: area under the curve
CI: confidence intervals
IRIS: International Renal Interest Society
KD: kidney disease
MRS: medullary rim sign
MRS‐*band*: medullary rim sign associated with a thick ill‐defined hyperechoic band
MRS‐*line*: medullary rim sign associated with a thin well‐defined hyperechoic line
N: cats without kidney disease
NoMRS‐KD: cats without medullary rim sign with kidney disease
NoMRS‐N: cats without medullary rim sign without kidney disease
ROC: receiver operating characteristic
Sn: sensitivity
Sp: specificity
US: ultrasonographic
US: ultrasound
USG: urinary specific gravity

## 1 INTRODUCTION

On ultrasound (US) examination, the medullary rim sign (MRS) is defined as a distinct hyperechoic line in the renal medulla, parallel to the corticomedullary junction.^1^, ^2^ It has been described in dogs with both acute and chronic kidney disease (KD) such as hypercalcemic nephropathy, chronic interstitial nephritis, acute tubular necrosis,^1^, ^3^, ^4^ and in dogs with no signs of renal dysfunction.^2^ A hyperechoic area between the cortex and the medulla, corresponding to the outer medulla is considered normal in dogs, especially in small breeds, and should not be interpreted as a rim sign.^5^ Additionally, a hypoechoic band in the corticomedullary junction in kidneys with a hyperechoic cortex and medulla, referred to as a “halo sign,” also has been reported in some dogs and cats with ethylene glycol toxicity.^4^, ^6^ This sign has been associated with a poor prognosis.^4^, ^6^

Medullary rim sign is a common US finding in healthy cats of all breeds.^7^ Furthermore, a hyperechoic line in the outer medulla has been associated with a band of mineral deposits in patients without KD.^8^ However, the MRS and the halo sign also have been described in cats with KD such as pyogranulomatous vasculitis associated with feline infectious peritonitis and chronic interstitial nephritis.^1^ In a recent study, MRS was identified in both azotemic and nonazotemic cats.^9^ Another recent study reported the prevalence and clinical relevance of the MRS in cats.^10^ Its clinical relevance however still remains unclear. In another study, both presence of MRS and visualization of a thick MRS were associated with KD.^10^ In addition, some confusion regarding the definition of MRS persists. We hypothesized 2 forms of MRS appearance may exist: a physiologic thin line and a pathologic thick band. Our aim was to describe the US appearance of MRS in cats and to assess its association with the presence of KD.

## 2 MATERIALS AND METHODS

### 2.1 Case selection criteria

For this retrospective case‐control study, the electronic medical records of all cats examined at the Veterinary Teaching Hospital of the University of Bologna between June 2008 and May 2017 were reviewed. Cats were included if the keyword “rim sign” appeared in the US report, if US images of the urinary tract were available for review, and if clinical and laboratory data (CBC, serum biochemistry and urinalysis) at the time of US examination were available. Cats were excluded if they underwent partial US examination or had incomplete images of the urinary tract or renal parenchymal changes preventing visualization of the MRS (eg, lymphoma, polycystic kidney disease) or urinary tract obstruction.

The MRS patients selected were divided into 2 groups: cats with KD (group MRS‐KD) and cats without KD (group MRS‐N).

Cats were diagnosed with KD based on the presence of compatible history, clinical, laboratory and US findings. In particular, included cats must have had either persistent azotemia (serum creatinine concentration >1.6 mg/dL) or persistently low urine specific gravity (USG <1.035), both assessed and confirmed over 1‐month as well as an US report consistent with KD.^11^ In this way, cats with International Renal Interest Society (IRIS) stage 2, 3 and 4 were included in the KD group. Patients without these abnormalities were included in the cats without KD disease (N) group.

The control group was selected by reviewing medical records of cats that underwent US examination during the same time period (2008‐2017). Cats were included in the cats without medullary rim sign with kidney disease (NoMRS‐KD) group if they met the criteria for KD (as defined above) without visualization of a MRS and included in the cats without medullary rim sign without kidney disease (NoMRS‐N) if they had neither evidence of KD nor MRS. Division of the control and study groups is summarized in Table 1.

**TABLE 1:** Flow chart illustrating the division of the control and study groups

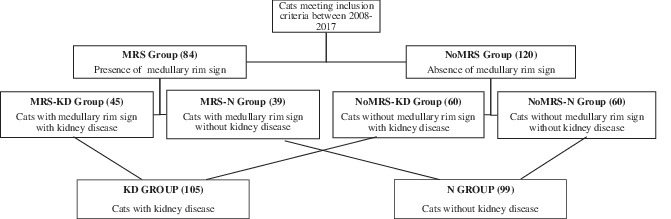

### 2.2 Ultrasound image review

All US images of the kidneys were randomly and blindly reviewed by a board‐certified radiologist unaware of the clinical diagnosis and US report findings. Recorded US findings included presence or absence of MRS and whether the MRS was unilateral or bilateral and if a thin hyperechoic line with well‐defined margins (MRS‐*line*) or a thick hyperechoic band with ill‐defined margins (MRS‐*band*) was present. A thin hyperechoic line was defined as a line approximately 1 mm in thickness with echogenicity greater than that of the adjacent medulla. A thick hyperechoic band was defined as band with a thickness >2 mm. Margins were considered ill‐defined when the contours of the band were indistinct. Renal size was evaluated by measuring length in dorsal scan.^12^, ^13^ Renal contours (smooth or irregular) were evaluated in 3 planes; kidneys with a normal bean shape and smoothly delineated capsule were considered as having smooth contours whereas kidneys with focal concave or convex distortion of the capsule and loss of normal bean shape were considered as having irregular contours. Corticomedullary distinction (good or poor; and when poor, decreased or absent) was subjectively evaluated. It was judged good if clear distinction was present between the hypoechoic medulla and the more echogenic renal cortex. When corticomedullary distinction was considered poor, it was further categorized as decreased if it was still visible but subjectively less conspicuous than normal or absent if there was no distinction between the 2 zones. Presence of mineral foci casting acoustic shadows in the peridiverticular recesses, nephroliths or both (present or absent) was recorded. Pelvic distension (present or absent; if present, with dimensions in mm) was evaluated in the transverse plane as previously described,^13^ and echogenicity of the perirenal tissue (normal or abnormal) also was recorded.

### 2.3 Statistical analyses

Normal distribution of data was assessed by means of the D'Agostino‐Person test. Data were reported as mean and SD or median and range (minimum and maximum values), based on distribution. Differences among groups for continuous variables were evaluated using the Mann‐Whitney *U* test or the Kruskall‐Wallis test with compensated post hoc analysis. Frequencies of the alterations in the US variables evaluated in the study (renal contours, corticomedullary distinction, mineral foci in the peridiverticular recesses, nephroliths, pelvic distension, and perirenal tissue) were compared among the study groups using Fisher's exact test or a chi‐squared test. The diagnostic accuracy of each US finding to distinguish cats with KD from cats without KD was evaluated using the receiver operating characteristics (ROC) curve. Sensitivity (Sn) and specificity (Sp) were reported for each US finding, as well as the area under the curve (AUC), reported with its 95% confidence intervals (CI). The value of the AUC as a criterion of accuracy was considered as follows: low, 0.5 to 0.7; moderate, 0.7 to 0.9; and high, >0.9.^14^ All statistical analyses were performed using commercially available statistical software (MedCalc Statistical Software version 19.0.7, MedCalc Software bvba, Ostend, Belgium). Significance was set for a *P* value <.05.

## 3 RESULTS

### 3.1 Animals

Based on our inclusion criteria, 84 cats (MRS group) were included in the study: 39/84 cats (46%) had no sign of KD (MRS‐N group), whereas 45/84 (54%) had signs of KD (MRS‐KD group).

The 2 control groups (NoMRS‐KD and NoMRS‐N) consisted of 60 cats each.

Signalment of the cats included in each group are summarized in Table 2. Selected laboratory data comparing cats with a MRS with and without KD are presented in Table 3.

**TABLE 2:** Signalment of the cats included in the control and study groups

	MRS‐KD	MRS‐N	NoMRS‐KD	NoMRS‐N
Groups	(n = 45)	(n = 39)	(n = 60)	(n = 60)
Age^a^	10.8 (1‐16.5)	7.8 (0.5‐15)	8.5 (2.5‐15.5)	7.3 (0.5‐14)
Males (N)	28 (22)	21 (18)	32	31
Females (S)	17 (15)	18 (14)	28	29
Breed: DSh	37	31	52	50
Per	3	2	2	3
Sia	2	1	2	0
MCo	0	2	2	4
NFo	1	1	2	1
Aby	0	1	0	1
Sph	1	0	0	1
Cha	1	0	0	0
Bur	0	1	0	0

Abbreviations: Aby, Abyssinian; Bur, Burmese; Cha, Chartreux; DSh, Domestic Shorthair; MCo, Maine Coon; MRS‐KD, cats with medullary rim sign and kidney disease; MRS‐N, cats with medullary rim sign without kidney disease; N, neutered; NFo, Norwegian Forest Cat; NoMRS‐KD, cats without a medullary rim sign with kidney disease; NoMRS‐N, cats without a medullary rim sign without kidney disease; Per, Persian; S, spayed; Sia, Siamese; Sph, Sphynx.

a Age presented as median and range (minimum‐maximum).

**TABLE 3:** Selected laboratory data comparing cats with a medullary rim sign with (MRS‐KD) and without (MRS‐N) kidney disease

	MRS group (N = 84)			
	MRS‐KD (N = 45)		MRS‐N (39)	
	N	Median (range)	N	Median (range)
USG	27	1.020 (1.011‐1.035)	22	1.052 (1.024‐1.080)
Serum creatinine (mg/dL)	40	2.7 (1.6‐30.8)	30	1.3 (0.6‐1.6)
Serum urea (mg/dL)	43	88 (29.7‐710.7)	36	49.2 (20.7‐105.9)
Total calcium (mg/dL)	42	9.5 (1.2‐11.4)	34	9.7 (7.7‐11.3)

Abbreviation: USG, urine specific gravity.

### 3.2 Ultrasound findings

All US examinations were performed using ultrasound units (iU22 ultrasound system, Philips Healthcare, Monza, Italy; Epiq ultrasound system, Philips Healthcare, Monza, Italy) equipped with probes of different frequencies. For the examination of the urinary system, both microconvex (8‐5 MHz) and linear array (12‐5, 16‐5 MHz) probes were used. The prevalence of MRS in our population of cats (all cats underwent US examination of the abdomen between 2008‐2017) was 4.6%.

**FIGURE 1:**
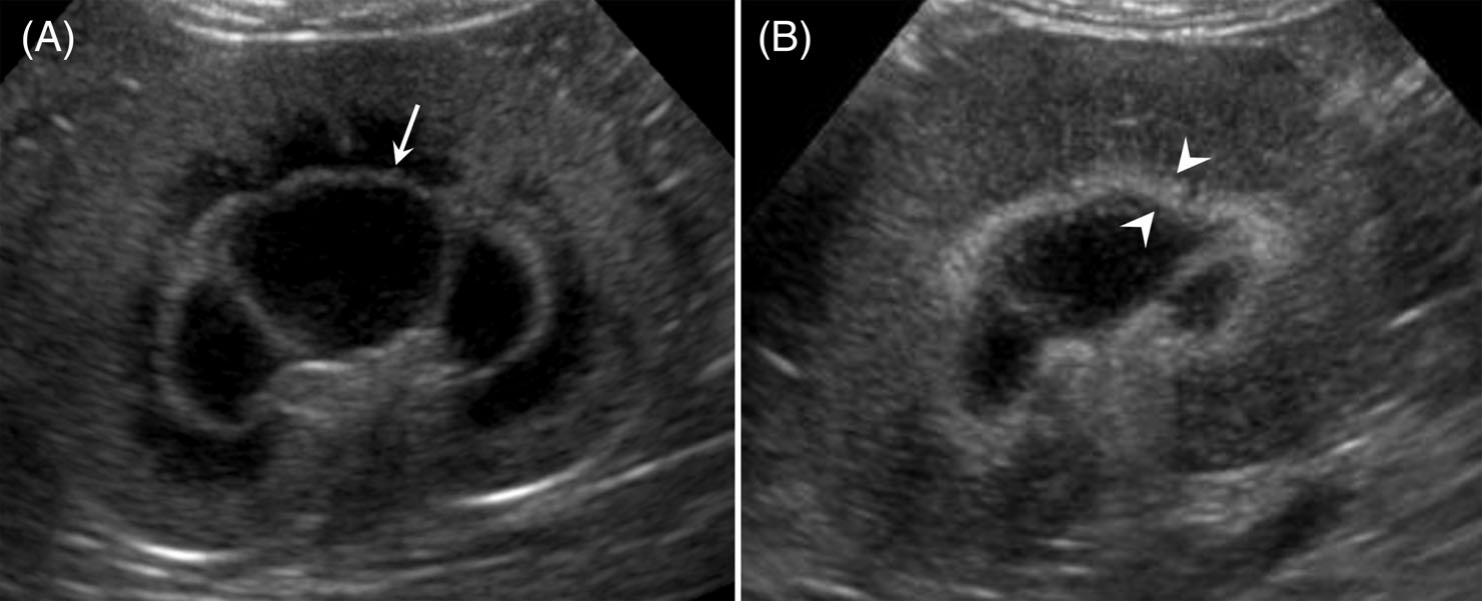
Representative ultrasonographic images from two cats included in the study, showing medullary rim sign (MRS) in the renal parenchyma (microconvex probe 8‐5 MHz). A, Dorsal image of the left kidney of a cat without sign of kidney disease (group MRS‐N). Note the feature of MRS characterized by a thin hyperechoic well‐defined line, MRS‐*line* (white arrow). B, Dorsal image of the left kidney of a cat with sign of kidney disease (group MRS‐KD). The MRS appeared as thick hyperechoic ill‐defined band, MRS‐*band* (between white arrowheads)

In all but 1 case (83/84 cats; 99%) MRS was bilateral. The MRS appeared as a thin hyperechoic line with well‐defined margins (MRS‐*line*) in 50/84 (59%) cats (Figure 1A); 30/50 (60%) did not have KD, whereas 20/50 (40%) had KD. These results are presented in Table 4.

**TABLE 4:** Flow chart illustrating the division of the study groups (MRS group) according to the aspect of the MRS and number of patients recruited in each group

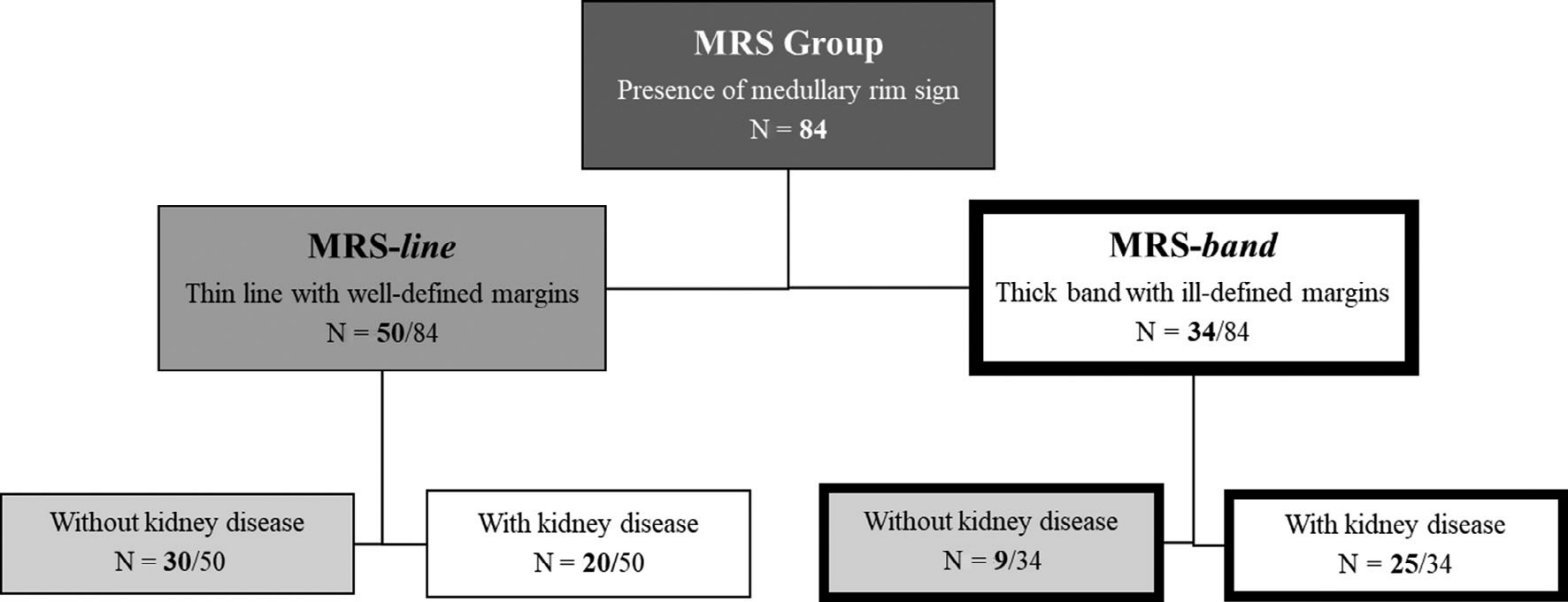

*Notes:* MRS‐*line*: thin hyperechoic well‐defined line; MRS‐*band*: thick hyperechoic ill‐defined band.

In the remaining 34/84 cats (41%), the MRS appeared as a thick hyperechoic band with ill‐defined margins (MRS‐*band*; Figure 1B); 25/34 cats (74%) had signs of KD (Table 4).

**FIGURE 2:**
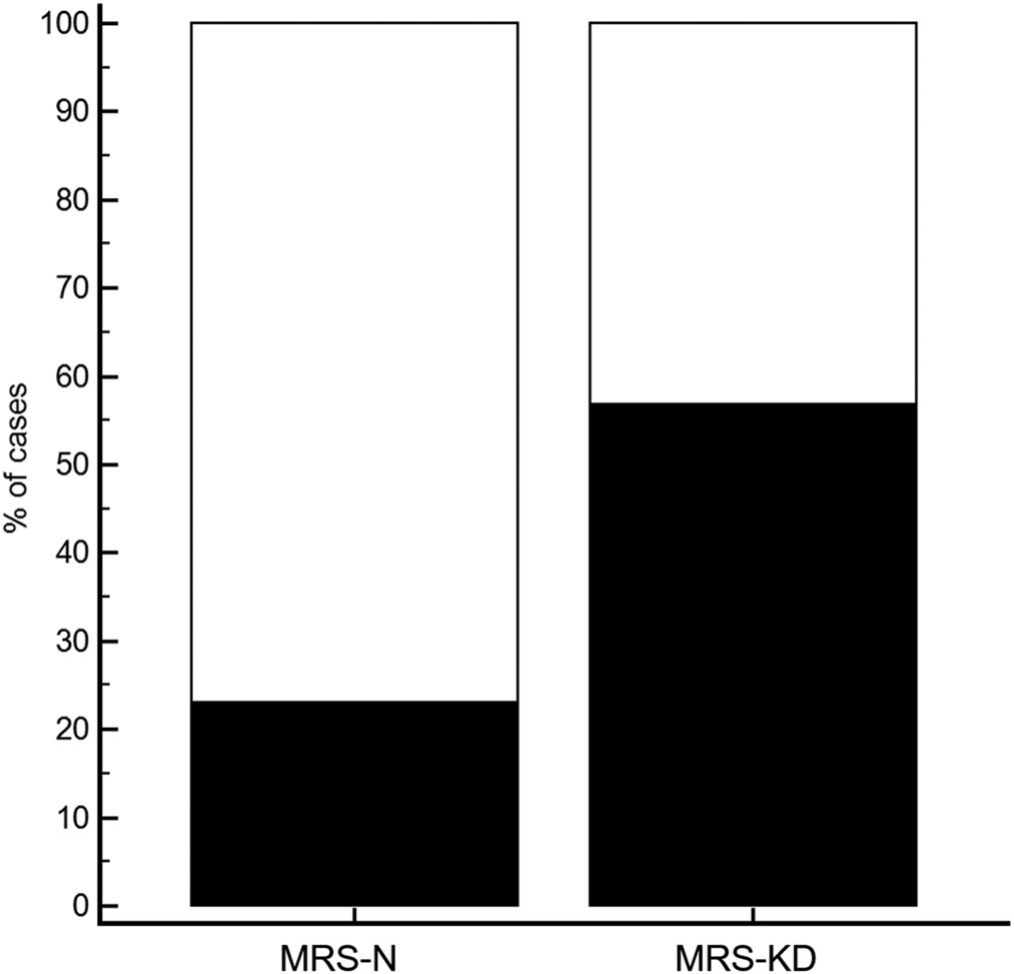
Graph depicting the frequency of the thin hyperechoic line with well‐defined margins (white boxes), MRS‐*line* and the thick hyperechoic band with ill‐defined margins (black boxes), MRS‐*band* in 39 cats with medullary rim sign without sign of kidney disease (MRS‐N group) and in 45 cats with medullary rim sign with kidney disease (MRS‐KD group). Thin hyperechoic line (MRS‐*line*) was mainly observed in cats without renal disease, while thick hyperechoic band (MRS‐*band*) was more frequent in cats with renal disease (*P* = .003)

In the MRS‐N group, 30/39 (77%) cats had an MRS‐*line* and 9/39 (23%) cats had an MRS‐*band* (Figure 2). An MRS‐*line* was significantly more frequent in cats without KD, whereas presence of an MRS‐*band* was significantly more frequent in cats with KD (*P* = .003).

When comparing the group with an MRS‐*line* and the group with an MRS‐*band*, no statistically significant difference was seen regarding the age of the cats (*P* = .31).

In total, 105 cats had KD (KD group) and 99 cats did not have KD (N group).

**FIGURE 3:**
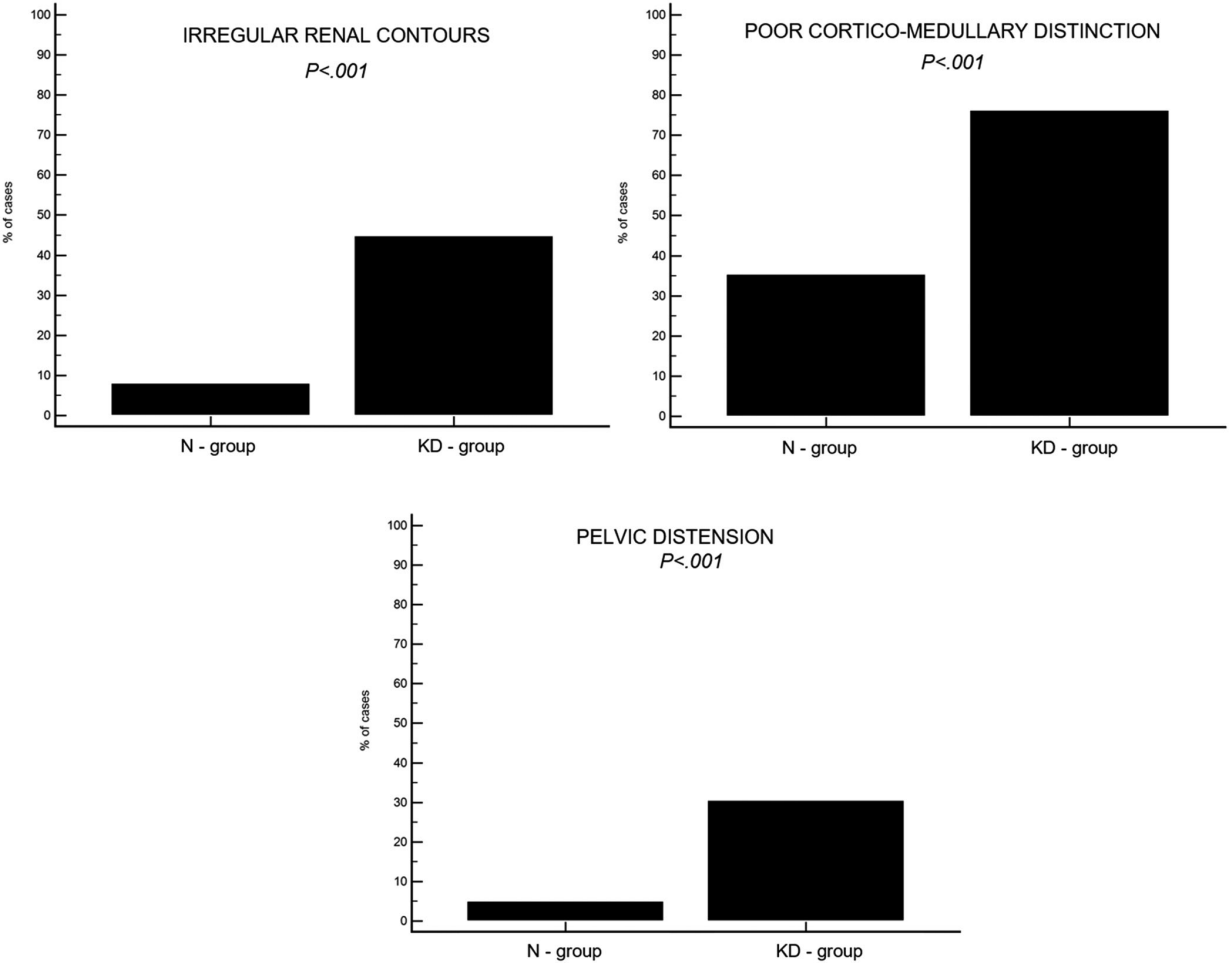
Graphs showing the frequency of the different ultrasonographic findings (ie, irregular renal contours; poor corticomedullary distinction, pelvic distension) in 99 cats without kidney disease (N‐group) and in 105 cats with kidney disease (KD‐group)

No significant difference was found in renal length for either kidney among the 4 groups. All other findings (ie, renal contour, corticomedullary distinction, mineral foci in the peridiverticular recesses, nephroliths, pelvic distension, and perirenal tissue) were more frequent in the groups of cats with KD, when considering all groups combined (Figure 3).

Poor corticomedullary distinction differed within the N group, being significantly more frequent in cats from the MRS‐N group than in cats from the NoMRS‐N group (*P* < .001).

Other US signs for the 4 groups are summarized in Table 5.

**TABLE 5:** Results of the comparison of ultrasonographic findings in the four groups of cats included in the study

Ultrasonographic findings	MRS‐KD (n = 45)	MRS‐N (n = 39)	NoMRS‐KD (n = 60)	NoMRS‐N (n = 60)	*P* value
Left kidney length (mm)	37.0 (25.0‐52.0)	38.0 (31.0‐46.0)	37.0 (18.0‐71.0)	38.6 (26.2‐50.2)	.17
Right kidney length (mm)	38.0 (12.0‐52.0)	39.0 (29.0‐47.0)	37.0 (18.0‐71.0)	38.8 (25.9‐49.3)	<.001
Pelvic distension (mm)	0 (0‐6)	0 (0‐4)	0 (0‐19)	0 (0‐1)	<.001
Irregular and bumpy contours (yes)	12/45 (27%)	2/39 (6%)	35/60 (58%)	6/60 (10%)	<.001
Poor corticomedullary distinction (yes)	35/45 (78%)	24/39 (62%)	45/60 (75%)	11/60 (18%)	<.001
Mineral foci (yes)	3/45 (7%)	1/39 (3%)	23/60 (38%)	5/60 (8%)	<.001
Nephroliths (yes)	2/45 (4%)	1/39 (3%)	10/60 (17%)	2/60 (3%)	.01
Pelvic distension (yes)	9/45 (20%)	1/39 (3%)	23/60 (38%)	4/60 (7%)	<.001
Altered perirenal tissue (yes)	2/45 (4%)	0/39 (0%)	14/60 (23%)	3/60 (5%)	.009

*Notes:* Data are reported as median and range (minimum‐maximum value) or frequency and percentage of total cases. A *P* < .05 was considered significant. Differences among groups for continuous variables (kidney length and pelvic distention): Mann‐Whitney *U* test/Kruskall‐Wallis test with compensated post hoc analysis; frequencies (renal contours, corticomedullary distinction, mineral foci in the peridiverticular recesses, nephroliths, pelvic distension, and perirenal tissue): Fisher exact test/chi‐squared test. *P* value refers to the difference between the 4 groups.

Abbreviations: MRS‐KD, cats with medullary rim sign and kidney disease; MRS‐N, cats with rim sign and without kidney disease; NoMRS‐KD, cats without rim sign and with kidney disease; NoMRS‐N, cats without rim sign and without kidney disease.

The most accurate variable to distinguish cats with KD, identified by ROC curve analysis, was corticomedullary distinction, with AUC = 0.712 (0.648‐0.777 95% CI), followed by renal contour, with AUC = 0.683 (0.629‐0.738 95% CI) and pelvic distension, with AUC = 0.627 (0.578‐0.676 95% CI; Table 6).

**TABLE 6:** Results of the receiver operating characteristic (ROC) curve analysis for the discrimination between 105 cats with kidney disease (KD) and 99 cats without kidney disease (N)

Variable	AUC	95% CI	Se (%)	Sp (%)	+LR	−LR	*P* value
Irregular and bumpy contours (yes/no)	0.683	0.629‐0.738	44.8	92.9	5.54	0.60	<.001
Poor corticomedullary distinction (yes/no)	0.712	0.648–0.777	76.2	64.6	2.16	0.37	<.001
Mineral foci (yes/no)	0.594	0.546‐0.624	24.8	93.9	2.44	0.70	<.001
Nephroliths (yes/no)	0.542	0.507‐0.577	11.4	97.0	3.77	0.91	.02
Pelvic distension (yes/no)	0.627	0.578–0.676	30.5	94.9	6.04	0.73	<.001
Altered perirenal tissue (yes/no)	0.561	0.522‐0.599	15.2	97.0	5.03	0.87	.002

*Notes:* Values of best sum of sensitivity and specificity are reported. A *P* value <.05 was considered significant. Differences between cats with KD and N: ROC curve.

Abbreviations: AUC, area under the receiver operating characteristic curve; CI, confidence interval for AUC; Se, Sensitivity; Sp, Specificity; +LR, positive likelihood ratio; −LR, negative likelihood ratio; KD, cats with kidney disease; N, cats without kidney disease.

## 4 DISCUSSION

In this retrospective case‐control study, we confirmed the presence of 2 distinct categories of MRS (ie, MRS‐*line* and MRS‐*band*) and an association between the US appearance of the MRS and the presence of KD in cats. A thin hyperechoic line with well‐defined margins and a thick hyperechoic band with ill‐defined margins were the 2 distinct types of MRS observed. The frequency of the MRS‐*line* was higher in cats without KD, whereas the MRS‐*band* was significantly more frequent in cats with KD. In another study,^10^ the presence of an MRS was associated with KD, whereas in our study, no association was found between MRS and KD. Although both studies had the similar objective to investigate the clinical relevance of MRS in cats there were some substantial differences between the studies. In our study, all US images of the kidneys were randomly and blindly reviewed by a board‐certified radiologist, unaware of the clinical diagnosis and US findings, which was not the case in the previous study, where selection bias may have been introduced.^10^ Despite these differences, the 2 studies agreed on the most relevant observation and concluded that the presence of a MRS‐*band* was associated with KD.

The underlying cause of the appearance of an MRS is currently under investigation and not fully understood. The MRS‐*line* may be the result of an area of intraluminal mineral deposits within renal tubules in patients without KD as described previously.^8^ Unfortunately, no histological examination of the kidneys was available in our study, making the confirmation of mineral deposits impossible.

Ours is the second report describing a thick band with ill‐defined margins, which may lead to confusion between its appearance and that of a thin medullary rim line. An MRS‐*line* was significantly more frequent in cats with KD, making it an important variable to consider when evaluating cats with MRS. A different histopathological mechanism for formation may be involved other than benign mineral deposits.^8^ We hypothesize that a main mechanism involved may be vascular in origin, because the area in which the band is located corresponds to the outer medulla, a substantially more hypoxic region, even in normal kidneys.^15^, ^16^ Intrarenal oxygen availability is the balance between supply, mainly dependent on renal blood flow and demand, determined by metabolic needs. Renal blood flow is carefully maintained to ensure stable glomerular filtration, and therefore increased intrarenal oxygen consumption can lead to tissue hypoxia.^15^ Tubulointerstitial hypoxia stimulates production of collagen and smooth muscle actin resulting in increased fibrogenesis. Furthermore, the hypoxic environment induces epithelial‐mesenchymal transdifferentiation thus worsening fibrosis, and resulting in decreased peritubular perfusion and oxygen delivery because of capillary rarefaction.^15^ On US examination, fibrosis, collagen, and smooth muscles fibers appear generally hyperechoic. For these reasons, we hypothesize that the presence of a thick hyperechoic band in the outer medulla may be the consequence of increased fibrogenesis because of chronic tubulointerstitial hypoxia, possibly enhanced by the age. Renal histopathology would have been necessary to confirm this hypothesis.

We hypothesize 2 distinct pathogeneses for the MRS‐*line* and MRS‐*band*, and the new term medullary band sign (MBS) is proposed to describe the most likely pathologic condition. The use of different terminology (ie, MRS for a thin well‐defined line and MBS for thick ill‐defined band) to describe 2 different signs with 2 possible clinical meanings could clarify the US description and be useful from a clinical point of view.

Cats with KD were older than cats without KD, which was expected considering that the prevalence of the KD increases with age and is higher in geriatric patients.^7^, ^9^, ^17^, ^18^

Surprisingly, the MRS‐KD group showed a higher median age with respect to the remaining groups; no relation is known between age and the presence of MRS. Although no statistical difference was found between the age of the cats with MRS and MBS, it is possible that age plays a role in the appearance of MRS in cats, and additional studies would be necessary to rule out or confirm this hypothesis.

In almost all cases except for 1 cat, the MRS was bilateral. This cat showed a marked difference in renal size: the right kidney was substantially smaller than the left kidney and had a completely altered US appearance because of atrophy, which may have prevented visualization of the MRS.

No significant differences were found regarding the dimensions of the kidneys in the 4 groups, both for the left and the right side. This observation is in agreement with results of a recent study, in which no difference was found in mean renal length between azotemic and nonazotemic cats.^9^

In our study, the most reliable variables for distinguishing cats with KD from cats without KD were poor corticomedullary distinction, irregular contours, and pelvic distension in cats with or without MRS (overall population). This result may have been affected by the number of cats with MRS included in the study, but the number of cats in each group was similar and adequate for comparison. Increased echogenicity of the cortex was not retained as a relevant US criterion for KD, as it has been found that the echogenicity of the cortex in cats can be affected by different factors, such as presence of fat vacuoles in the cortical tubular epithelium or technical factors such as frequency and type of transducer.^8^, ^19^ According to a recent study, a hyperechoic cortex is the most frequent US alteration in nonazotemic cats.^9^

As previously described, median pelvic diameter was higher in cats with KD. The variable degree of pelvic distension in cats with KD can be explained by fluid administration, as observed in dogs, secondary to polyuria or partial functional or mechanical ureteral obstruction.^9^, ^20^

Poor corticomedullary distinction was significantly more frequent in cats with KD. Surprisingly, when considering the 2 groups without KD (MRS‐N and NoMRS‐N groups), poor corticomedullary distinction appeared more frequent in cats with MRS. We can hypothesize that the subjective evaluation of this US sign can be affected by the presence of a hyperechoic line in the renal parenchyma that artifactually mimics cortical thickening, creating visual illusion, possibly due to an optical phenomenon.

Although more frequently observed in cats with KD, mineral foci in the peridiverticular recesses, nephroliths or both also were seen in cats without KD. Criteria used for the diagnosis of KD in cats in our study may not have been sufficiently sensitive, and these US signs may reflect subclinical renal disease at the time of US examination.^9^, ^16^, ^21^ In addition, symmetric dimethyl arginine was not systematically evaluated in our cats and may have helped identify cats with earlier onset KD.^11^

A previous study reported an association between presence of a MRS and final diagnosis of feline infectious peritonitis, where a thin, marked intensity MRS was identified (although the association with KD was described for thick MRS).^10^ In our study population, no cats had a diagnosis or suspicion of feline infectious peritonitis.

The main limitations of our study were a consequence of its retrospective nature. We included cats in the study group on the basis of the term rim sign in their US reports. In this way, some cases may have been missed, because some radiologists may not have reported its presence, because he or she may have assumed it was not clinically relevant. The prevalence of MRS in our population might be underestimated, and this possibility also could explain the lower prevalence obtained compared to the prevalence observed in a previous study (36%).^10^ It was not possible to further characterize the type of KD and correlate MRS with the chronicity of KD. In addition, cats with IRIS stage 1 KD were not identified in our study, because of the inclusion criteria required for the diagnosis, which may have led to the inclusion of some of these patients in the N groups. In addition, both convex (8‐5 MHz) and linear (12‐5, 16‐5 MHz) transducers were used. Type of transducer employed also can affect renal echogenicity,^8^, ^19^, ^22^ and this factor can be considered a potential limitation of our study. Furthermore, a single radiologist reviewed the images and categorized the type of MRS according to qualitative and semiquantitative criteria. Another limitation is lack of renal histopathology to further define the origin of the MRS.

In conclusion, MRS was observed in both cats with and without KD. A thin hyperechoic well‐defined line (MRS‐*line*) was more frequent in cats without KD, whereas a thick hyperechoic ill‐defined band (MRS‐*band* or so‐called MBS) frequently was associated with KD. Presence of an MBS in association with poor corticomedullary distinction, irregular contours, and pelvic distension is US evidence of KD in cats.

## CONFLICT OF INTEREST DECLARATION

Authors declare no conflict of interest.

## OFF‐LABEL ANTIMICROBIAL DECLARATION

Authors declare no off‐label use of antimicrobials.

## INSTITUTIONAL ANIMAL CARE AND USE COMMITTEE (IACUC) OR OTHER APPROVAL DECLARATION

Authors declare no IACUC or other approval was needed.

## HUMAN ETHICS APPROVAL DECLARATION

Authors declare human ethics approval was not needed for this study.
